# The Impact of Storms on *Legionella pneumophila* in Cooling Tower Water, Implications for Human Health

**DOI:** 10.3389/fmicb.2020.543589

**Published:** 2020-12-10

**Authors:** Robin L. Brigmon, Charles E. Turick, Anna S. Knox, Courtney E. Burckhalter

**Affiliations:** Savannah River National Laboratory, Environmental Science and Biotechnology Group, Aiken, SC, United States

**Keywords:** *Legionella pneumophila*, cooling tower, weather, biocide, water chemistry

## Abstract

At the U.S. Department of Energy’s Savannah River Site (SRS) in Aiken, SC, cooling tower water is routinely monitored for *Legionella pneumophila* concentrations using a direct fluorescent antibody (DFA) technique. Historically, 25–30 operating SRS cooling towers have varying concentrations of *Legionella* in all seasons of the year, with patterns that are unpredictable. Legionellosis, or Legionnaires’ disease (LD), is a pneumonia caused by *Legionella* bacteria that thrive both in man-made water distribution systems and natural surface waters including lakes, streams, and wet soil. Legionnaires’ disease is typically contracted by inhaling *L. pneumophila*, most often in aerosolized mists that contain the bacteria. At the SRS, *L. pneumophila* is typically found in cooling towers ranging from non-detectable up to 10^8^ cells/L in cooling tower water systems. Extreme weather conditions contributed to elevations in *L. pneumophila* to 10^7^–10^8^ cells/L in SRS cooling tower water systems in July–August 2017. *L. pneumophila* concentrations in Cooling Tower 785-A/2A located in SRS A-Area, stayed in the 10^8^ cells/L range despite biocide addition. During this time, other SRS cooling towers did not demonstrate this *L. pneumophila* increase. No significant difference was observed in the mean *L. pneumophila* mean concentrations for the towers (*p* < 0.05). There was a significant variance observed in the 285-2A/A Tower *L. pneumophila* results (*p* < 0.05). Looking to see if we could find “effects” led to model development by analyzing 13 months of water chemistry and microbial data for the main factors influencing the *L. pneumophila* concentrations in five cooling towers for this year. It indicated chlorine and dissolved oxygen had a significant impact (*p* < 0.0002) on cooling tower 785A/2A. Thus, while the variation in the log count data for the A-area tower is statistically greater than that of the other four towers, the average of the log count data for the A-Area tower was in line with that of the other towers. It was also observed that the location of 785A/2A and basin resulted in more debris entering the system during storm events. Our results suggest that future analyses should evaluate the impact of environmental conditions and cooling tower design on *L. pneumophila* water concentrations and human health.

## Introduction

*Legionella pneumophila*, the cause of Legionnaires’ disease (LD), is a Gram-negative bacterium ubiquitous in man-made and natural aquatic environments, where it survives in biofilms (Wery et al., 2008). Legionellosis, or Legionnaires’ disease, is a pneumonia caused by *Legionella* bacteria that thrive both in man-made water distribution systems and natural surface waters including lakes, streams, and wet soil (Fliermans, 1985). Legionnaires’ disease is typically contracted by inhaling *Legionella* bacteria, most often in aerosolized mists that contain the bacteria (Ishimatsu et al., 2001). Pontiac fever is generally a nonfatal respiratory disease caused by various species of Legionella that has resulted in outbreaks of influenza-like diseases (Ward et al., 2010).

There are currently over 60 known *Legionella* species and 71 serogroups of *L. pneumophila*, with many of these serogroups implicated in human disease (Me′rault et al., 2011; Katsiaflaka et al., 2016; Miyashita et al., 2020). *L. pneumophila* serogroup 1 is most frequently implicated in disease and is the strain most commonly found in the natural environment (Marston et al., 1994; Lawrence et al., 1999; Lettinga et al., 2002). However, other Legionella types have also been implicated in disease, most often *Legionella pneumophila* serogroups 4 and 6, as well as other serogroups (Buchbinder et al., 2002; Fields et al., 2002; Paranjape et al., 2020), and coinfections with several *Legionella* species due to immunodeficiencies (Matsui et al., 2010).

Most world-wide LD outbreaks have been associated with cooling towers, since *Legionella* can persist in these systems and release viable bacteria in the aerosolized effluents (Berendt, 1980; Sanchez et al., 2008). Under certain conditions, including low disinfectant concentrations, high organic levels, and warm temperatures, *Legionella* can readily propagate in these structures (Kusnetsov et al., 1997). In recent years, it is likely that more cases of LD have been reported worldwide due to increased awareness of *Legionella* in the environment and workplace, more accurate and accessible clinical testing, technological advances in microbial source tracking, and increased monitoring of water treatment systems (Fields et al., 2002). Current clinical methods for detecting *Legionella* antigen in the urine of infected patients targets primarily Serogroup 1 (Bibb et al., 1984; Aguero-Rosenfeld and Edelstein, 1988). The *Legionella* urinary antigen test has had a major impact on epidemiological trends in outbreaks of Legionellosis, as it circumvents the difficulty of clinical isolation and identification (Alvarez et al., 2009). It is possible that some species have not yet been associated with human disease because they occur so rarely in nature; therefore, all *Legionella* strains should be considered potentially pathogenic (Atlas, 1999). Since *Legionella* species are fastidious to isolate and culture, identification of new species/serogroups is exceedingly difficult (Hussong et al., 1987; Bentham, 2000).

The detection or presence of *Legionella* in water or on a fixture or device is not enough to cause disease (Kim et al., 2002). For disease to develop, individuals must inhale enough virulent organisms to overwhelm their natural resistance (Shelton et al., 2000). A common source of *Legionella* is from cooling towers, commonly used for cooling water, with utility ranging from industrial processes to residential buildings. Towers with excess organics including pollen, soil, and other detritus; and other suspended solids, such as algae, and other microorganisms can shield *Legionella* from biocides and provide an environment conducive to bacterial growth (Pinel et al., 2020). *Legionella*bacteria must be virulent and, in enough concentrations, to cause disease (Marston et al., 1994). In most cases, the water source must be aerosolized and distributed so the human host can inhale *Legionella*through mists (Nguyen et al., 2006). For disease to occur, *Legionella* must be inhaled or aspirated deeply into the lungs to the alveoli by the potential host(s), and the host must be physiologically unable to stop the infection (Dalebroux et al., 2009). The infectious dose of *Legionella* for humans has not been determined, but the larger the dose, the more likely an infection will occur (Emmerson, 2001). Length of exposure can also be a factor and the risk of infection is greater when the dose of *Legionella*-containing water is in direct, close contact with the susceptible population, as is the case with humidifiers/foggers or maintenance work on water systems (Berendt, 1980; Bentham and Broadbent, 1993). Meteorological conditions conducive to low-level inversion and high humidity have been linked to cooling tower LD outbreaks and/or to *Legionella* stability in aerosols (Ishimatsu et al., 2001; Dunn et al., 2013). Cooling towers near workers or the general population are of concern because of the potential exposure from massive amounts of aerosolized mists generated (Brown et al., 1999).

In epidemiological studies of *Legionella* disease outbreaks, the infection rates have been demonstrated to be greater for those who are hospitalized, older, smokers, heavy drinkers, disease compromised, or on immunosuppressive therapy, and these factors are often listed as risk factors for LD. Other factors that may contribute to higher LD risk are diabetes, chronic bronchitis, cancer, AIDS, and end-stage renal disease. It is theorized that a lower dose of *Legionella* may cause infection in individuals with one or more of these conditions (Fields et al., 2002).

Occupational sources of *Legionella* include building, industrial, and other man-made water systems. These manufactured water systems can include cooling towers, scrubbers, evaporative condensers, humidifiers, water heaters, holding tanks, pipes, shower heads, faucets, nebulizers, misters, and whirlpool baths (Cooper et al., 2004). Groundwater sources for municipal use and other water distribution systems have been found to contain *Legionella* spp. (Costa et al., 2005). *Legionella* bacteria survive in low numbers in routinely treated domestic water and can be carried into buildings through domestic treated water. The bacteria can colonize and be transmitted from plumbing fixtures, including shower heads and hot-water taps (Bollin et al., 1985; Allegra et al., 2020). Investigations of nosocomial infections in hospitals have often found the cause to be the potable water supply. Cooling towers and industrial water systems have been shown to be colonized with *Legionella* through the make-up water.

Cooling towers have been the principal agents for disseminating *Legionella* with resulting disease and, in some cases, deaths (Alvarez et al., 2009). Most cases of Legionellosis occur as sporadic cases, not epidemics. The risk exists not only for people in the buildings and structures where the cooling towers are located, but also to passers-by and people some distance from the tower who might be exposed to cooling tower mists. In a 1978 outbreak, people were infected both inside and outside a hospital near the source cooling tower (Dondero et al., 1980). In that case, a correlation was demonstrated between LD cases and areas of the hospital that received ventilating air from intakes close to the cooling tower containing *L. pneumophila.*

The concentration of *Legionella* that has been linked to the causation of disease associated outbreaks is as low as 2.3 Colony Forming Units (CFU), depending on the source and conditions (Whiley et al., 2014). For example, an outbreak in Sweden was linked to a *Legionella* concentration of 1.2 × 10^9^ cells/L in cooling tower water (Ulleryd et al., 2012). “Health-threatening” levels of *Legionella* have been determined to be 1.0 × 10^6^ cells/L culturable populations, while “high *Legionella* concentration” has been defined as 1.0 × 10^7^ cells/L, and “high level of concern” has been defined as ranges from 10^8^ to 10^9^ cells/L concentrations (Atlas, 1999).

A variety of biocides used alone or in combination are used for *Legionella* control, including chlorine (Cl), bromine (Br), ozone, and various formulations (Kim et al., 2002; Miller and Koebel, 2005). Effective biocide chemistry encompasses both the primary element in the biocide (Cl, Br, etc.) and maintenance of water quality (e.g., conductivity). Effective biocide use demands maintenance of prescribed concentrations and the adjustment of concentrations to changing demand associated with elevated temperatures or heavy organic concentrations. Temperature can impact *L. pneumophila* growth in cooling tower biofilms and circulating water as well as the associated microbial and eukaryotic communities (Paniagua et al., 2020). Maintenance issues, including scale and dust build up, can influence biocide activity, and adjusting biocide concentration to account for these influences may enhance *Legionella* control. Corrosion or deterioration of water tower construction materials can lead to increased metal concentrations in circulating water. Biocides can also cause corrosion in water distribution as well (Marchesi et al., 2016.) The results of microbiological studies indicate that while elevated concentrations of certain metals are toxic, lower levels of iron, zinc, and potassium can enhance *L. pneumophila* growth (Zahran et al., 2017). While ozone has been shown to be an effective biocide when there are no organics, its use offers no residual activity and control. The use of specific biocides depends on the needs of the tower, the environmental regulations on effluents, specific design of the cooling tower, need for biofilm penetration, and cost efficiency (Lau and Ashbolt, 2009). Material in water distribution systems is also a consideration in selection of chemical treatment, since some biocides can be more corrosive than others (Marchesi et al., 2016). The aim of this study is to understand the impact of water chemistry and seasonality on *L. pneumophila* in cooling towers operating under a variety of conditions in a temperate location. A similar approach may be applied to other cooling towers in different geographic locations.

## Materials and Methods

**FIGURE 1:**
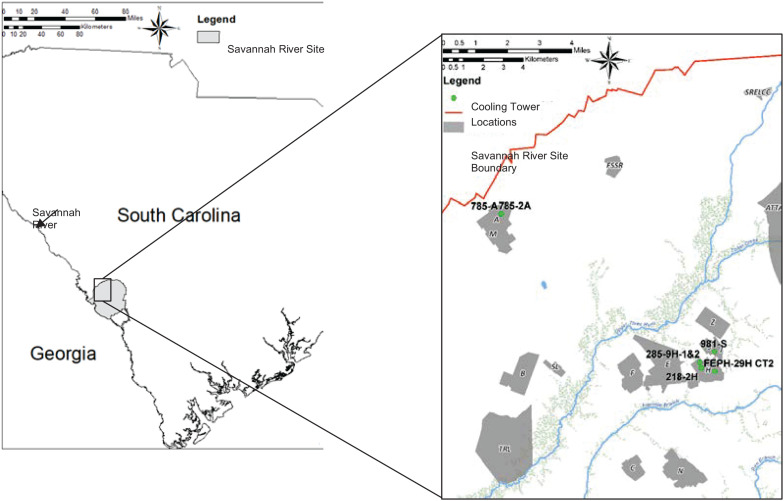
Geographical distribution of cooling towers 785-A/785-2A, 285-9H-1&2, 218-2H, FEPH-29H CT2, and 981-S relative to the Savannah River Site in South Carolina.

At the U.S. Department of Energy’s Savannah River Site (SRS) in Aiken, SC, cooling tower water is routinely monitored for *L. pneumophila* concentrations (serogroups 1, 2, 4, and 6) on a monthly or quarterly basis using a direct fluorescent antibody (DFA) technique. Historically, the 30 operating SRS cooling towers have had varying concentrations of *Legionella* in all seasons of the year, with patterns that are unpredictable. The cooling towers are of varying age, water treatment system, construction, size, water supply, and geographical distribution over the 320 square miles of the SRS. A stoplight system based on cooling tower water *L. pneumophila* concentrations has been developed to help operators control microbial growth. For this system, “green” is from 0 to 10^6^ cells/L, “yellow” is 10^6^–10^7^ cells/L, and “red” is 10^7^ cells/L or greater. Red requires extra vigilance by management and biocide shocking procedures to bring *L. pneumophila* concentrations down. Extreme weather conditions contributed to elevations in *L. pneumophila* up to 10^7^–10^8^ cells/L in the SRS cooling tower water systems. The location of the five cooling towers used in this work relative to the SRS are shown in Figure 1.

### Water Samples

Sterilized 500 mL polycarbonate sample bottles are used to sample these individual cooling tower waters. Water samples are collected directly from tower basins and split into two 500 mL samples. One sample is placed in a cooler and the other is used for on-site analysis for physical and chemical parameters. A calibrated Yellow Springs Instrument (YSI) MPS 552 multi-parameter meter (YSI, Yellow Springs, Ohio) is used to measure temperature (°C), conductivity [Siemens per meter (S/m) in SI and millimhos per centimeter (mmho/cm)], pH, and dissolved oxygen (DO) (%) on site after collection. Palintest 1000 test kits are used to measure total Br and/or free Cl (Palintest House, Kingsway, United Kingdom).

### Microbiological Analysis

*Legionella pneumophila* is concentrated in the cooling tower water samples (500 mL) in the laboratory by filtering water samples through a filtration membrane (47-mm-diameter, 0⋅4-μm pore size filters-Whatman Nucleopore; GE Healthcare Life Sciences, Piscataway, NJ, United States) prior to DFA testing. Each filter set was aseptically cut and placed into a 15-mL conical tube (Thermo Fisher Scientific), 1 ml of 0⋅2-μm-filter sterilized FA Buffer (Difco; Thermo Fisher Scientific, Detroit, MI, United States) was added, and tubes were vortexed for 4 min. Eight-well glass slides (Carlson Scientific, Peotone, IL, United States) pretreated at 90°C were rinsed with 70% ethanol prior to sample deposition. Inactivated *L. pneumophila* serogroups 1, 2, 4, and 6 cells were acquired from Monoclonal Technologies, Inc. (Alpharetta, GA, United States) and served as positive controls for the DFA. *Serratia marcescens* (ATCC 13880) served as the DFA negative control well as a water control to test for non-specific binding. Sample replicates (10 μL) were added to four wells of a prepared slide and heat fixed at 80–90°C for 10–15 min. Slides were then placed into a 25°C humidified chamber for 20 min. Each heat fixed sample was separately stained with 20 μL of antibody fluorescein isothio-cyanate (FITC)-labeled monoclonal antibodies for *L. pneumophila* serogroups 1, 2, 4, and 6 (Monoclonal Technologies, Inc.). These serogroups were selected as they are most commonly linked with disease outbreaks, especially serogroup 1 (Shelton et al., 1994; Fields et al., 2002). Slides were rinsed with DI water and stored overnight in FA buffer at 25°C. Slides were then rinsed with 5% sodium pyrophosphate (Difco; Thermo Fisher Scientific) buffer and allowed to dry in the dark at 25°C. Slides were then examined and DFA labeled *L. pneumophila* cells counted with fluorescent microscopy (Zeiss Axioscope 2) at 1000× and concentrations of the species and serotype determined as previously described (Leskinen et al., 2012). The positive and negative controls were run with each set of monthly cooling tower samples.

### Statistical Analysis

Comparisons of the *L. pneumophila* concentrations (cells/L) for a 13-month period between 2016 and 2017 with physical, chemical, and environmental parameters were performed with JMP Pro Version 11.2.1 SAS Institute Inc., Cary, NC, 2014. These parameters included cooling water pH, conductivity (mS/cm), temperature (water and air) (°C), DO (%), Cl, Br, turbidity (NTU), wind (m/s), and rain (in). Five SRS cooling towers that were sampled monthly during this time period were selected for analysis including 218-2H, 285-9H-1&2, FEPH-29H-CT3, 981-S, and 785A/2A. Cooling tower 785A/2A was sampled two times monthly when *L. pneumophila* concentrations were elevated for a total of 15 replicates.

## Results

**FIGURE 2:**
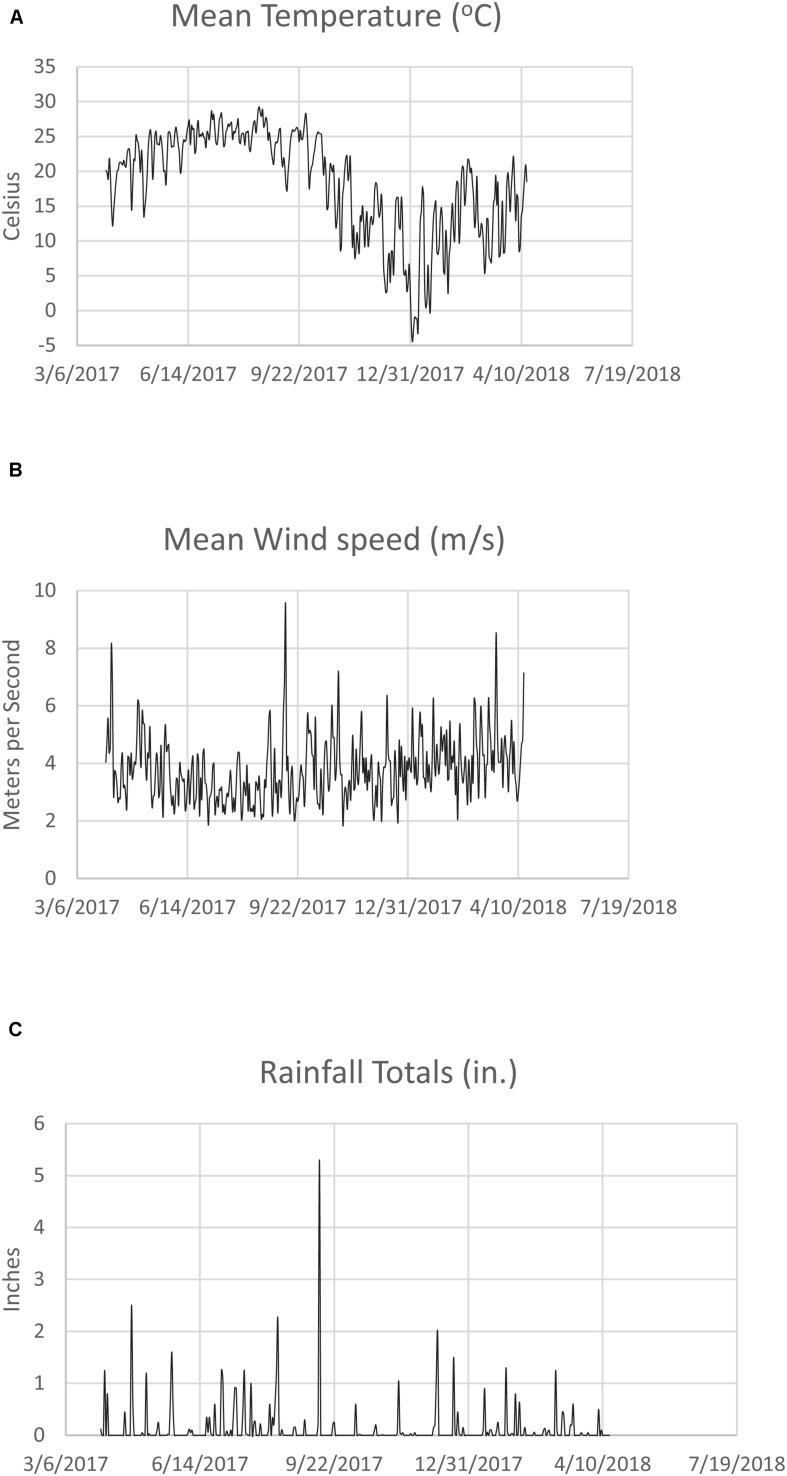
**(A)** Mean monthly Savannah River Site temperature (°C) for 3/6/2017–7/19/2018. **(B)** Mean monthly Savannah River Site wind speed (m/s) for 3/6/2017–7/19/2018. **(C)** Mean monthly Savannah River Site rainfall (in.) for 3/6/2017–7/19/2018.

Mean monthly Savannah River Site temperature (°C), windspeed (m/s), and rainfall total (in.) for 4/1/2017–4/15/2018 is shown in Figures 2A–C, respectively. Note the peak rainfall and wind speeds for August and September 2017, when Hurricanes Harvey and Irma came through the Southeastern United States. Temperature was slightly down during this time period due to the storm conditions (wind, rain, clouds). While the Savannah River Site was not directly hit by these hurricanes, the wind and rain had a major impact. On September 11, 2017, wind speed peaked at 9.51 m/s (Figure 1B), and rainfall was 5.30 inches in one day (Figure 1C). In a nationwide study, a precipitation threshold above 750 mm (2.95 inches) was found to contribute significantly to elevated legionellosis activity (Han, 2019).

**FIGURE 3:**
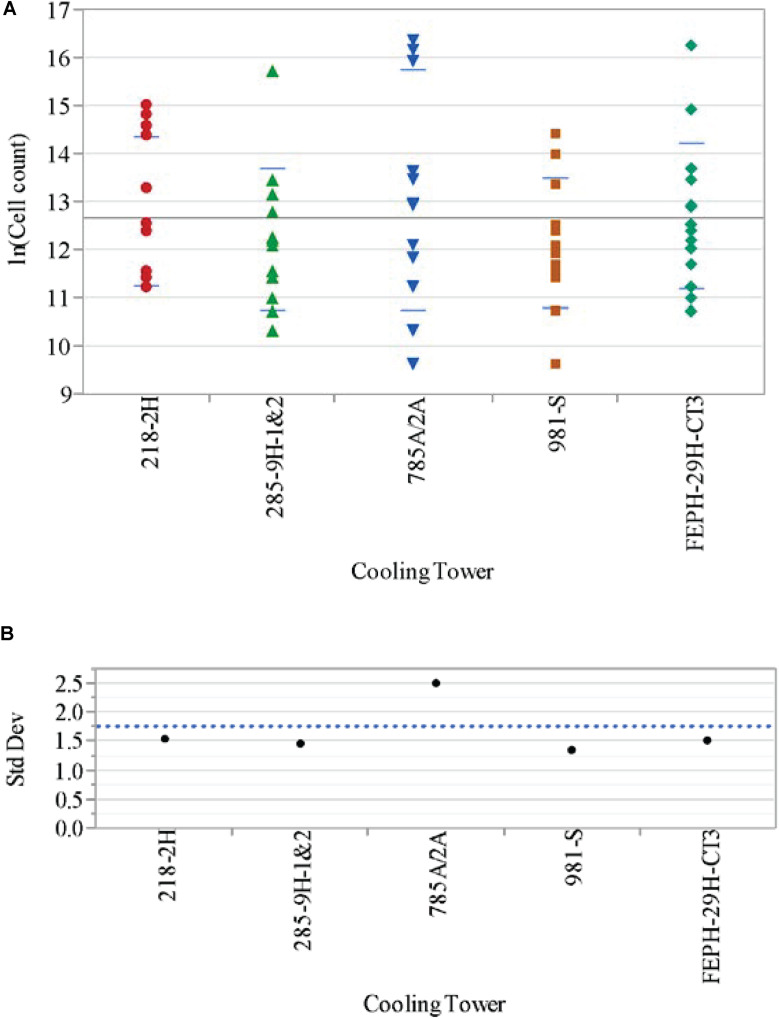
**(A)** Analysis of *Legionella pneumophila* concentrations ln(Cell count) by cooling tower. **(B)** Variance of *Legionella pneumophila* concentrations ln (cell count) by cooling tower.

Comparing the data across the cooling towers for the *L. pneumophila* concentrations (expressed in natural logs, i.e., ln[L A (cells/L)]) yielded the results shown in Table 1. Included in Table 1 are the standard deviations (std devs) in each cooling tower, as well as the results for Levene’s and Welch’s statistical tests. Levene’s test for variance equality for the log count data for *L. pneumophila* across the cooling towers indicates that the variances are statistically different at the 5% significance level, with 785A/2A having the largest variance. Figure 3A shows the analysis of ln (Cell count) by cooling tower for the year, demonstrating the variance in A-Area Tower.

**TABLE 1:** Cooling tower water *Legionella pneumophila* concentration comparisons.

Level	Count	Std dev	MeanAbsDif to Mean	MeanAbsDif to Median
218-2H	12	1.543120	1.345350	1.304034
285-9H-1&2	12	1.463006	1.044911	1.038666
785A/2A	15	2.501282	2.064472	2.044219
981-S	12	1.340178	0.992456	0.985878
FEPH-29H-CT3	14	1.521459	1.125929	1.098230

**Test**	**F ratio**	**DFNum**	**DFDen**	**Prob > *F***

O’Brien[.5]	3.3175	4	60	0.0160
Brown-Forsythe	2.4468	4	60	0.0560
Levene	2.8055	4	60	0.0334
Bartlett	1.7714	4	.	0.1314

**Welch’s Test**				

Welch Anova testing Means Equal, allowing Std Devs Not Equal				
**F ratio**	**DFNum**	**DFDen**	**Prob > *F***
0.7932	4	29.83	0.5390

Figure 3B provides a plot of the variances, showing the higher deviation in the A-Area Tower. The results from Welch’s test for the means of the cooling tower *L. pneumophila* monthly data being equal indicates that there is no statistically significant difference (at the 5% significance level) in the means of these data (Table 1). Thus, while the variation in the log count data for the A-area tower is statistically greater than that of the other towers, the average of the log count data for the A-area tower is in line with that of the other towers.

### Tests That the Variances Are Equal

**FIGURE 4:**
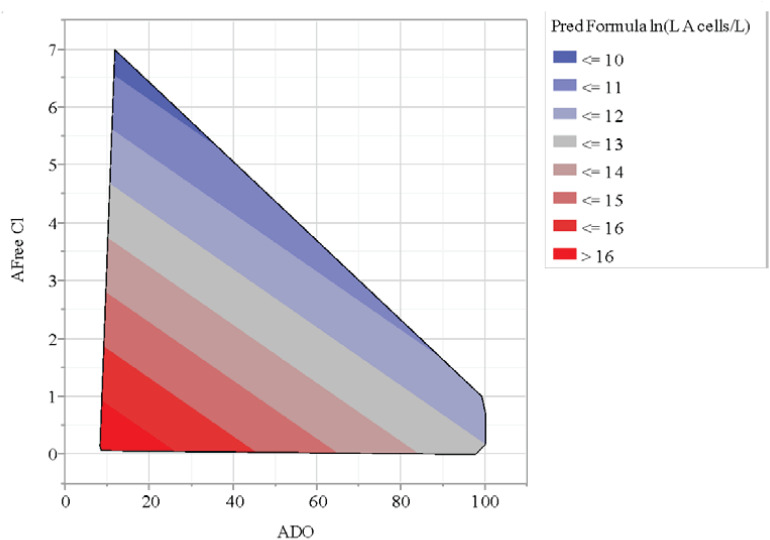
Contour plot of chlorine (Cl) and dissolved oxygen (DO) relationship with *L. pneumophila* concentrations (cells/L).

A statistical model of the ln[L A (cells/L)] values for the A-Area Tower was explored. The results of this effort are in Table 2. The model for ln[L A (cells/L)] as a linear function of DO and Free Cl (as measured in A Tower) explained 86.4% of the variation seen in these log data with no indication of a lack of fit for the model. Both parameter estimates are negative, which implies that as DO and/or Free Cl increases, the model predicts that the *L. pneumophila* concentrations are expected to decrease. The contour plot of model predictions shows this graphically in Figure 4. It was found that Br cooling tower water concentrations, generally 50% lower than Cl, were not significant regarding *L. pneumophila* concentrations. Similarly, the cooling tower water pH averaged around 9.0, but was also found to be not significant with respect *to L. pneumophila* concentrations.

**TABLE 2:** Analysis of variance for Cooling Tower 785-2A dissolved oxygen (DO) and free chlorine (Free Cl) relative to *Legionella pneumophila* concentrations.

Summary of Fit				
RSquare				0.861
R Square Adj				0.837
Root Mean Square Error				1 106
Mean of Response				13.183
Observations (or Sum Wgts)				13

**Analysis of Variance**								

**Source**	**DF**	**Sum of Squares**	**Mean Square**	**F Ratio**

Model	2	77.754	38.877	31.804
Error	10	12.224	1.222	Prob > F
C. Total	12	89.978		< 0.0001^∗^
**LackOfFit**								
Lack Of Fit	8	10.5978	1.325	1.629
Pure Error	2	1.626	0.813	Prob > F
Total Error	10	12.224		0.435
				Max RSq

**Parameter Estimates**								

**Term**	**Estimate**	**Std error**	***t* Ratio**	**Prob > |*t*|**

Intercept	17.427	0.625	27.90	<0.0001*
ADO	−0.052	0.007	−7.00	<0.0001*
AFree Cl	−1.039	0.181	−5.75	0.0002*

*Significant at the 5% level.

**FIGURE 5:**
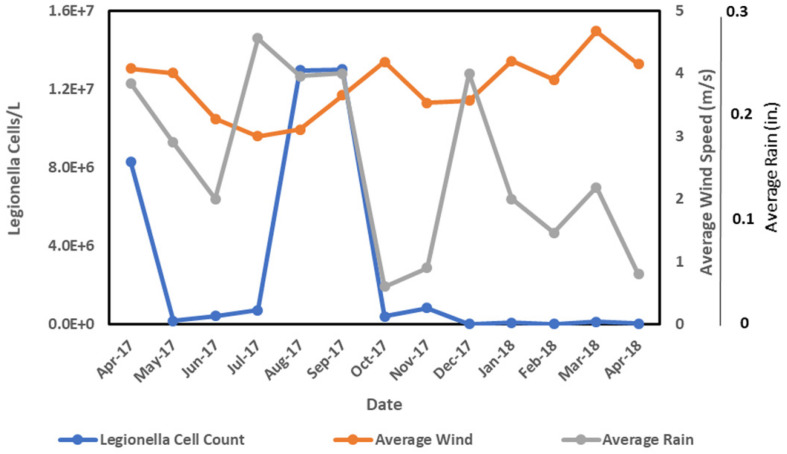
Average wind and rain effects on *Legionella* concentrations for the 785A/2A cooling tower.

Environmental conditions can impact *L. pneumophila* control in cooling towers, as observed with extreme 2018 summer weather in data presented here. Cooling tower 785-A/2A concentrations went from averaging 10^5^–10^6^ cell/L to 10^7^–10^8^ cell/L after Hurricanes-Harvey-and-Irma-associated extreme weather (Figure 5). The increase in *L. pneumophila* concentrations seemed to lag just behind the heavy rain, indicating a cumulative effect (Figure 5). Despite automated biocide addition, these increases were likely due to impact of windblown debris into the cooling tower and basin with excessive rain. The towers are typically cleaned once yearly during the winter months, so regular maintenance was not a factor in this case. This increase of debris observed in the cooling tower basin may have contributed to the decrease in DO associated with the increase in *L. pneumophila* concentrations (Figure 4). It has been shown that monitoring *Legionella* aerosols around contaminated cooling towers during fog conditions may be a valuable contribution to the risk assessment and prevention of LD outbreaks (Villanueva and Schepanski, 2019). For more efficient monitoring for *Legionella* detection, new experimental predictive methods of contamination could be implemented alongside the classical microbiological methods, such as Geostatistics, which could be applied just in the case of aerosol distribution from the cooling towers (Laganà et al., 2015; De Giglio et al., 2019; Allegra et al., 2020). An experimental setup provided data that demonstrated *Legionella* risk exposure and associated dose-response of a *Legionella* infection from aerosols generated by nebulizers to lung deposition (Allegra et al., 2020).

## Discussion

Contamination of man-made water distribution systems including water supplies with *Legionella* is an established cause of legionellosis. Water sources of large buildings, such as hospital distribution systems and cooling towers, are often contaminated with *Legionella* and therefore represent a potential danger to patients, occupants, and workers (Brown et al., 1999; Wellinghausen et al., 2001). Efficient water monitoring and detection of elevated *L. pneumophila* concentrations is essential to prevent cases from occurring (Buchbinder et al., 2002; Chang et al., 2009; Carducci et al., 2010). While *L. pneumophila* is the main causative agent of legionellosis, detection and differentiation can be difficult due to problems with nonviable cultures (Edagawa et al., 2009). *L. pneumophila*-containing aerosols from anthropogenic devices, including cooling towers, are known to be a problem with a direct impact on human health (Allegra et al., 2020), Because *legionellae* can be difficult to culture and cells may be damaged due to biocides as well as the desire for direct identification, immunofluorescence is often preferred for identification and quantification (Bibb et al., 1984; Edelstein and Edelstein, 1989). In a previous study at SRS with the same DFA applied here, cooling tower water concentrations of *Legionella* were determined using a Portable Multi-use Automated Concentration System (PMACS) concentrates micro-organisms from large volumes of water through automated dead-end ultrafiltration and backflushing (Leskinen et al., 2012). The DFA is a rapid test that does require expertise as whole cells are counted to determine concentrations. While live cells can be determined alongside DFA with a counter stain, that was not done here. The sensitivity of DFA has been found to be about 70% for detection of *L. pneumophila* serogroup 1 with specificity approaching 99% (Pierre et al., 2017). The DFA technique has been a standard for our laboratory for determination of *L. pneumophila* in cooling towers (Fliermans, 1985). For cooling tower water, the DFA allows rapid analysis for samples with varying complicating factors including high biocides, e.g., Cl and other chemicals including anticorrosion agents. DFA results from SRNL cooling tower water have been correlated with qPCR findings in past unreported studies with Clemson University (Tamara McNealy, personal communication).

*Legionella pneumophila* is known to thrive in certain natural aquatic environments without biocides including rain puddles (Sakamoto et al., 2009). Cl can react with organic matter such as the debris observed in the cooling tower basin and leave behind unwanted compounds such as trihalomethanes (THMs) and haloacetic acid (HAAs), which reduce Cl effectiveness (Al-Abri1 et al., 2019). While biocides, in this case Cl, have long been used to control *L. pneumophila* growth in water distribution systems (Marchesi et al., 2016) the interesting factor here is the interaction with DO. The correlation of DO and Cl- is of interest as *L. pneumophila*, a microaerophilic microorganism, has been found to typically thrive in water DO of 6.0–6.7 mg/L (Wadowsky et al., 1985). In that work *L. pneumophila* did not replicate in tap water which contained less than 2.2 mg of DO per liter. This particular cooling tower, 785A/2A, was subjected to rain, wind, and influx of debris into a basin that may have been conducive to biofilm formation. The large basin and build-up of debris set it apart from the other cooling towers evaluated that did not demonstrate elevated *L. pneumophila* concentrations during this time even through exposed to similar wind and rains from the extreme weather. While pH is known to impact *L. pneumophila* viability that prefers a near neutral pH (Wadowsky et al., 1985), it was not found to be a significant factor in the course of this 13-month study. Cooling tower operators often maintain the circulating water at a higher pH to limit corrosion.

Management of cooling towers can be a factor, as the presence of stagnant water, lack of maintenance, and/or environmental conditions can cause *L. pneumophila* buildup (Fliermans, 1985; Bentham, 2000). Interactions of *L. pneumophila* with amoeba can also complicate detection and eradication (McNealy et al., 2002). Amoebae can be infected with *L. pneumophila* in cooling towers that can vary greatly metabolically with different aquatic environments (Berk et al., 2006). *Acanthamoeba* have been found to contain respirable vesicles containing live *L. pneumophila* cells, indicating a unique survival mechanism for cooling tower environments, and may increase their survivability (Berk et al., 1998).

In this study we applied DFAs to four *L. pneumophila* known to cause most human disease cases. There are actually a variety of *Legionella* species that can cause LD (Miyashita et al., 2020). Many of these species due to their biodiversity are difficult to detect, isolate, and culture (Hussong et al., 1987). For this reason, the *Legionella* urinary antigen test has become the main tool for clinical diagnoses (Bibb et al., 1984). While *L. pneumophila* serogroup 1 causes from 50 to 80% of LD, it is estimated that as many as 20–50% of cases of LD are missed if the urine antigen is the only diagnostic test (Pierre et al., 2017).

Figure 5 demonstrates the impact of the elevated wind, rain, and subsequent increase in *L. pneumophila* concentrations. While rainfall was observed to be elevated in December, an increase in *Legionella* was not observed as rainfall was not prolonged as observed earlier in the July, August, and September time frame (Figure 5). Because the cooling tower water samples are taken monthly for *L. pneumophila* testing, the initial increase may have been earlier than the actual testing date. *L. pneumophila* outbreaks found to be from cooling tower sources have resulted in increased vigilance maintenance and control of existing cooling systems (Nguyen et al., 2006; Ulleryd et al., 2012). Legionnaires’ disease cases often remain largely underdiagnosed with outbreaks not properly determined, due to the pneumonia-like symptoms mimicking other ailments, and lack of testing (Spiegelman et al., 2020). In addition, while many biocides are available for water treatment and *Legionella* prevention, environmental aspects including other biota (Orrison et al., 1983; McNealy et al., 2002), water chemistry (Kim et al., 2002), and environmental factors can influence its survival and recalcitrance to biocides (Mallison, 1980). In Japan, *L. pneumophila* was found to be abundant in rainwater puddles, especially during warm weather (Sakamoto et al., 2009). These multiple sources could lead to higher background ambient levels of *L. pneumophila* due to anthropogenic aerosols generated by construction, traffic, or other activities (Parthuisot et al., 2010). Using the Nationwide Inpatient Sample and U.S. weather data, it has been estimated that the probability of community-acquired pneumonia (CAP) being diagnosed LD increases when weather is warm and humid (Simmering et al., 2017). The results were found to vary by region due to geographic and seasonal differences in humidity and temperature.

Biocides can react with other chemical species, rendering them ineffective. In the 2014–2015 LD outbreak in Flint, Michigan, it was found that an increase in free Cl demand, with increased concentrations of iron and assimilable organic matter from corroded pipes, stimulated *legionellae* growth and reacted chemically with free Cl, thereby reducing its biocide effectiveness (Zahran et al., 2017).

## Conclusion

While no cases of legionellosis were documented in this case at the SRS during this time of extreme weather, conditions including summer weather, high rainfall, increased humidity, and cloudy conditions were conducive for *Legionella* growth conditions. The factor of temperature and high precipitation have been linked with higher incidences of Legionellosis (Han, 2019). The potential for human exposure from cooling towers at these *Legionella* concentrations and atmospheric conditions are clear (Villanueva and Schepanski, 2019). A clean-out of the cooling tower basin and repeated biocide applications were required to bring *L. pneumophila* below 10^6^ cells/L –the safe or “green” level –in this cooling tower water. Other SRS towers, including the four in this comparison, were not exposed to as much debris as 785-A/2A. While they did not demonstrate the *L. pneumophila* increase despite the extra precipitation, and varied from not detected (ND) range up to 10^6^ cells/L as measured by DFA, the average for the year was the same; but significant variability in 785-A/2A was caused by the weather factors. While [Br] generally follows [Cl] in our cooling tower water monitoring, [Cl] alone was correlated with the *L. pneumophila* indicating water chemistry has to be closely followed in extreme conditions. Thus, the findings of this work emphasize the significance of understanding the resilience and ecology of *L. pneumophila* in cooling tower water in terms of public health.

Uncertainties related to *Legionella* risk assessment in cooling towers due to variations in operation, environmental conditions, and management have been discussed in this work. This is in part due to the fact source tracking can be difficult for legionellosis (Addiss et al., 1989). Biofilm development in cooling towers can also make control and detection of *L. pneumophila* in circulating water difficult (Declerck, 2010). The effectiveness of regulatory, monitoring, and health issues related to *Legionella* risk has been reviewed by Whiley et al. (2014). Recent findings of inhibitive solar effects of UV radiation and sunshine hours on legionellosis (Han, 2019) may help explain the increased level in *L. pneumophila* observed here under stormy and cloudy conditions.

In summary, we have shown that despite an operating biocide system, *L. pneumophila* concentrations were elevated in a cooling tower as a function of extreme weather conditions, including rain and wind bringing in debris. *L. pneumophila* concentrations in Cooling Tower 785-A/2A stayed in the 10^8^ cells/L range despite biocide addition due to environmental conditions. The cooling tower remained in the red zone containing higher cells/L than the permissible limit. Incorporation of free Cl and DO decreases the number of *L. pneumophila* in the cooling towers when possible. We have also shown that other towers were not as susceptible to weather conditions due to location and lack of a large basin. This issue of cooling towers and biocide and DO impact should be assessed on a case by case basis. Future work will include testing of *L. pneumophila* concentrations in cooling tower water with qPCR and culture techniques to asses viability and sensitivity factors.

## Data Availability Statement

All datasets generated for this study are included in the article/supplementary material.

## Author Contributions

All authors contributed equally to this manuscript in terms of sampling, testing, analysis, and authorship.

## Conflict of Interest

The authors declare that the research was conducted in the absence of any commercial or financial relationships that could be construed as a potential conflict of interest.
